# Multi-Epitope-Based Peptide Vaccine Against Bovine Parainfluenza Virus Type 3: Design and Immunoinformatics Approach

**DOI:** 10.3390/vetsci12111074

**Published:** 2025-11-09

**Authors:** Junbo Wang, Pu Wang, Fangyuan Tian, Qiang Liu, Meimei Hai, Zijie Guo, Yuanwen Wang, Yong Li, Yujiong Wang

**Affiliations:** 1 College of Life Sciences, Ningxia University, Yinchuan 750021, China; 12023131020@stu.nxu.edu.cn (J.W.); 12022140085@stu.nxu.edu.cn (P.W.); 12024130953@stu.nxu.edu.cn (F.T.); 12024140082@stu.nxu.edu.cn (Q.L.); 12023130991@stu.nxu.edu.cn (M.H.); 12023131012@stu.nxu.edu.cn (Z.G.); 12023131021@stu.nxu.edu.cn (Y.W.); 2 Key Laboratory of Conservation and Utilization of Biological Resources in Western China, Ministry of Education, Ningxia University, Yinchuan 750021, China; 3 School of Basic Medicine, Ningxia Medical University, Yinchuan 750004, China

**Keywords:** BPIV, subunit vaccine, F protein, HN protein, molecular docking

## Abstract

Bovine parainfluenza virus type 3 (BPIV3) is a significant pathogen implicated in bovine respiratory disease complex (BRDC), leading to lung tissue destruction, immunosuppression, and subsequent bacterial infections in cattle, hence incurring considerable economic losses globally. Notwithstanding its importance, a limited number of commercial vaccinations are presently accessible. The fusion (F) protein and hemagglutinin-neuraminidase (HN) protein, as protective antigens of the Paramyxoviridae family, can elicit neutralizing antibodies and are regarded as optimal candidates for the creation of genetically modified vaccines. A multi-epitope-based peptide vaccine (MEBPV) was developed by immunoinformatics methodologies by choosing epitopes from the F and HN proteins characterized by high antigenicity, moderate toxicity, and limited allergenic potential. The epitopes were combined with suitable linkers and adjuvants to produce the vaccine, whose physicochemical qualities, immunological attributes, solubility, and structural stability were improved and evaluated using computational methods. Molecular docking and molecular dynamics simulations demonstrated the strong potential binding affinity and stability of the vaccination with TLR2, TLR3, and especially TLR4 receptors. Immune simulations forecasted strong humoral and cellular responses, accompanied by a significant elevation in interferon-γ (IFN-γ) production. The vaccine sequence was later cloned into the pET-28a (+) vector for possible expression in Escherichia coli. Despite in silico predictions suggesting a favorable immunogenic potential, additional in vitro and in vivo studies are necessary to confirm its protective efficacy and safety. This research establishes a solid foundation for the creation of safe and efficacious subunit vaccines targeting BPIV3 and presents novel perspectives for the formulation of vaccinations against additional viral infections.

## Simple Summary

Bovine parainfluenza virus type 3 (BPIV3) is a significant pathogen responsible for bovine respiratory disease complex (BRDC), leading to pulmonary injury, immunosuppression, and secondary infections, hence incurring considerable economic losses in the cow sector. Existing commercial vaccines are inadequate and frequently offer insufficient protection, underscoring the necessity for innovative, safe, and effective vaccination tactics. A multi-epitope peptide vaccine (MEBPV) was designed using immunoinformatics methodologies, focusing on the fusion (F) and hemagglutinin-neuraminidase (HN) proteins. The vaccine exhibits advantageous physicochemical characteristics, structural integrity, immune receptor affinity, and anticipated immunogenicity, suggesting its potential as a new subunit vaccination candidate for BPIV3 infection prevention.

## 1. Introduction

Bovine parainfluenza virus type 3 (BPIV3) belongs to the Paramyxoviridae family and the Respirovirus genus [1]. It is an important pathogen responsible for respiratory illnesses in cattle, capable of infecting both calves and adult cattle [2]. BPIV3 contributes to the onset of bovine respiratory disease complex (BRDC) when co-infected with other pathogens, including bacteria or viruses. Infected calves display lung tissue damage, immunosuppression, and subsequent bacterial infections, leading to considerable economic losses for the global cattle industry [3].

BPIV3 was first isolated and reported in the United States in 1959 and has since been disseminated globally, exhibiting a pattern of universal prevalence. At present, BPIV3 is classified into three genotypes: A, B, and C. Genotype C was initially found in China and has become the major circulating genotype [4,5]. Research indicates that the C genotype strain possesses a high transmission capacity [6]. The genome of BPIV3 is approximately 15,000 nucleotides in length, encoding six structural proteins (N, P, L, M, F, and HN) and three non-structural proteins (C, D, and V) [7]. The fusion (F) protein and hemagglutinin-neuraminidase (HN) protein, as protective antigens of the Paramyxoviridae family, can stimulate the formation of neutralizing antibodies [7,8,9,10,11] and serve as ideal targets for the development of genetically engineered vaccines. The BPIV3 fusion (F) protein collaborates with the hemagglutinin-neuraminidase (HN) protein to promote membrane fusion, enabling the ingress of viral nucleic acid into the cytoplasm of host cells. Vaccines aimed at the F and HN proteins have been shown to effectively safeguard animals from viral infection [10,11,12,13].

In the absence of specific antiviral drugs, vaccination remains the primary strategy for the prevention and management of BPIV3. A systematic assessment of the literature up to September 2025 indicates that BPIV3 vaccine research has predominantly concentrated on live attenuated vaccines [14], recombinant chimeric vectors [15], DNA vaccines [16], recombinant protein or nanoparticle-based vaccines [10], and adenovirus-vectored vaccines [13]. Nevertheless, each method exhibits distinct constraints. Inactivated vaccines primarily induce humoral immunity, exhibiting little effectiveness in enhancing cellular immunity. Live attenuated vaccines may provide safety issues, including the risk of virulence reversion and genetic recombination between vaccine strains and wild-type strains [17]. Currently, few commercial vaccinations exist for BPIV3 [5,6]. In light of these problems, the prompt development of a safe and effective vaccine to prevent and control BPIV3 is essential. In contrast to traditional immunizations, multi-epitope peptide vaccines integrate numerous B- and T-cell epitopes, thereby addressing the restricted immunogenicity and poor immunological coverage associated with single-antigen vaccines [18,19,20]. They can provoke both humoral and cellular immune responses while diminishing allergenicity and toxicity [21,22]. Multi-epitope-based peptide vaccines have been extensively studied in various viruses, including HIV [23], SARS-CoV2 [24,25], HRV-C [26] and monkeypox virus (MPXV) [27]. Nonetheless, there are no documented instances of multi-epitope peptide vaccinations aimed at BPIV3.

**Figure 1:**
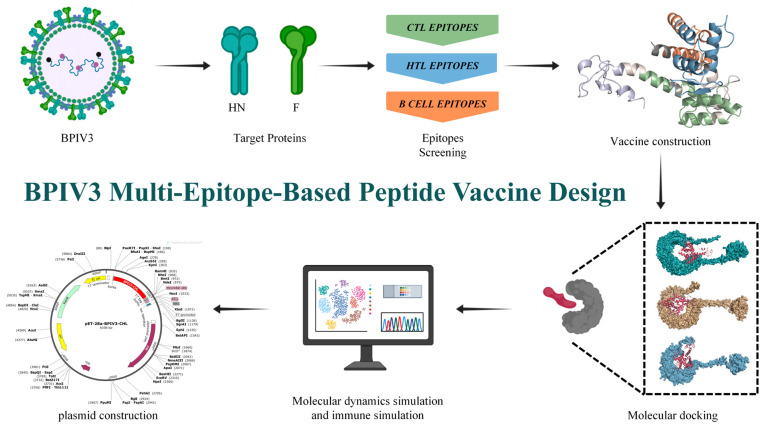
Flowchart illustrating the construction process of the multi-epitope-based peptide vaccine.BclI indicates a site flagged by SnapGene as potentially affected by DNA methylation or sequence context; plasmid DNA prepared from dam-/dcm- *E. coli* is expected to be cleavable at this site *.

In this study, we selected BPIV3 F and HN glycoprotein as protein targets, screened their immunodominant CTL, HTL, and B cell epitopes, and constructed a BPIV multi-epitope-based peptide vaccine through flexible serial connection. Then, the physicochemical properties, antigenicity, immunogenicity, toxicity, as well as the secondary and tertiary structures of the predicted multi-epitope-based peptide vaccine were analyzed and predicted. Subsequently, the binding affinity of the multi-epitope peptide vaccine to Toll-like receptors (TLRs) and its potential immunogenicity against BPIV were assessed by molecular docking, molecular dynamics simulations, and immunological simulation studies(Figure 1).

## 2. Materials and Methods

### 2.1. Obtaining Amino Acid Sequences

The amino acid sequences of the HN protein (ALS46557.1) and F protein (ALS46556.1) of bovine parainfluenza virus were obtained from the National Center for Biotechnology Information (NCBI) database (https://www.ncbi.nlm.nih.gov/, accessed on 2 April 2024) in FASTA format. Additionally, the amino acid sequence of β-defensin-3 (AAV41025.1) was also obtained from the NCBI database in FASTA format.

### 2.2. B Cell Epitope Prediction

B cell epitopes are crucial for eliciting humoral immunity and stimulating antibody production. Their amino acid composition interacts with the immune system to trigger antibody secretion. Recently, numerous computational tools have been developed to predict linear B cell epitopes, significantly advancing vaccine development and the design of immunological strategies [28]. In this study, we employed two tools for predicting linear B-cell epitopes. The first tool, Bepipred Linear Epitope Prediction 2.0, available through the Immunoepitope Database (IEDB) server (https://tools.iedb.org/bcell/, accessed on 5 April 2024), was used to identify the most prominent B-cell epitopes [29]. The second tool, ABCpred (https://crdd.osdd.net/raghava/abcpred/, accessed on 6 April 2024), employed a neural network-based methodology to forecast linear B-cell epitopes [30]. Predictions were conducted with a threshold exceeding 0.5 and a peptide length of 16 residues.

### 2.3. CTL Epitope Prediction

This study employed the NetMHCpan 4.1 EL method, as recommended by the IEDB (http://tools.iedb.org/mhci, accessed on 8 April 2024), to predict the binding affinity of CTL epitopes to MHC-I molecules. The tool’s machine learning algorithm was upgraded from NNAlign to NNAlign_MA to incorporate multi-allele MHC data [31]. Bovine MHC-I alleles available in the IEDB database were aligned with the BPIV3 protein sequence, and predictions below the percentile threshold (<0.05) were selected for further analysis. This stringent cutoff has been widely adopted in epitope-based vaccine design to identify strong binders, as lower percentile ranks correlate with higher binding affinity and better immunogenic potential [31,32].

### 2.4. HTL Epitope Prediction

The NetMHCIIpan 2.1 server (https://services.healthtech.dtu.dk/services/NetMHCIIpan-2.1/, accessed on 12 April 2024) was used to predict MHC-II epitopes [33]. This site utilizes artificial neural networks (ANNs) trained on a dataset including over 200,000 randomly chosen peptides [34]. It was selected for its inclusion of bovine leukocyte antigen (BoLA) MHC-II alleles, such as BoLA-DRB3, and its ability to target the HN and F proteins of bovine parainfluenza virus. It should be noted that the most recent releases of NetMHCIIpan do not fully support BoLA alleles, which limits their applicability for this study. Epitopes demonstrating a strong binding affinity to the selected MHC-II allele were identified using one of three criteria: binding affinity (IC50 < 50 nM), percentile rank (<0.5), or predicted score (>0.9). These thresholds are supported by benchmark studies demonstrating that peptides with IC50 < 50 nM or percentile ranks < 0.5% are strong binders, while predicted scores > 0.9 reflect high-confidence outputs from NetMHCIIpan models [35,36].

### 2.5. Epitope Evaluation

The antigenicity, sensitization potential, and toxicity of all predicted epitopes were evaluated using the VaxiJen v2.0 server (http://www.ddg-pharmfac.net/vaxijen/VaxiJen/VaxiJen.html, accessed on 17 April 2024) [37], AllerTOP v2.1 server (https://www.ddg-pharmfac.net/allertop_test/, accessed on 18 April 2024) [38], and ToxinPred server (http://crdd.osdd.net/raghava/toxinpred/, accessed on 19 April 2024) [39], respectively. Additionally, the IFNepitope server (http://crdd.osdd.net/raghava/ifnepitope/, accessed on 20 April 2024) was utilized to predict the ability of MHC-II epitopes to induce interferon-γ (IFN-γ), employing support vector machines (SVMs) and a mixture of models for analysis [40]. Immunogenic epitopes exhibiting high efficacy, devoid of toxicity or allergenicity, were chosen for the development of an effective vaccine.

### 2.6. Construction of Multi-Epitope-Based Peptide Vaccines (MEBPVs)

The multi-epitope-based peptide vaccine (MEBPV) was constructed by integrating cytotoxic T lymphocyte (CTL), helper T lymphocyte (HTL), and linear B lymphocyte (LBL) epitopes, in conjunction with suitable adjuvants and linker peptides. β-defensin-3 was chosen as the adjuvant owing to its reported ability to activate antigen-presenting cells through TLR1/2 signaling in studies [41]. While the immunogenicity and safety of defensins in cattle remain unverified, their evolutionary conservation and the demonstrated antimicrobial and immunomodulatory properties of bovine β-defensins indicate potential, necessitating further experimental validation within the bovine context [42].

CTL epitopes were connected using AAY linkers, HTL epitopes via GPGPG linkers, and LBL epitopes with KK linkers. These linkers are widely used for flexible separation of epitopes, minimizing junctional immunogenicity and facilitating proper epitope processing [43,44]. Although EAAAK linkers can increase structural rigidity, flexible linkers such as AAY, GPGPG, and KK were chosen to allow efficient antigen processing by proteasomes and lysosomes. Previous immunoinformatics investigations have emphasized the impact of linker length and flexibility on epitope presentation [45].

### 2.7. Physicochemical Properties and Solubility Assessment of Multi-Epitope-Based Peptide Vaccines (MEBPVs)

The ProtParam web server (https://web.expasy.org/protparam/, accessed on 28 April 2024) was used to predict the physicochemical properties of the vaccine, including molecular weight, instability index, amino acid composition, isoelectric point (pI), aliphatic index, and half-life [46]. The half-life is estimated using the “N-end rule,” linking protein stability to the N-terminal residue. The instability index (II) predicts overall protein stability based on dipeptide weights, with II < 40 indicating stability. The aliphatic index (AI) indicates the content of aliphatic side chains (Ala, Val, Ile, Leu) and their thermal stability. GRAVY represents the average hydropathy of all residues, indicating the protein’s hydrophobic or hydrophilic character. Immunological properties were evaluated using ToxinPred, AllerTOP v2.1, and VaxiJen v2.0. The solubility of the MEBPV was predicted using the Protein-Sol server (https://protein-sol.manchester.ac.uk/, accessed on 28 April 2024) [47].

### 2.8. Prediction, Optimization, and Validation of the Secondary and Three-Dimensional Structures of Multi-Epitope-Based Peptide Vaccines (MEBPVs)

The secondary structural characteristics of MEBPV, including β-turns, random coils, and α-helices, were predicted using the PSIPRED 4.0 server (https://bioinf.cs.ucl.ac.uk/psipred/, accessed on 5 May 2024). The PSIPRED 4.0 server executed protein secondary structure prediction with position-specific scoring matrices, achieving a prediction accuracy over 84% [48]. The tertiary structure of MEBPV was predicted using the Robetta server (https://robetta.bakerlab.org/, accessed on 13 May 2024) [49]. The Robetta server is a protein structure prediction platform built on the Rosetta macromolecular modeling suite [50]. To optimize the vaccine structure, the Galaxy Refine server was utilized. This server reconstructed all side chain clusters following side chain rearrangement perturbations and refined the structure through precise molecular dynamics (MD) simulations [51,52]. Subsequently, the stereochemical quality of the constructed structure was evaluated using the PROCHECK tool available on the SAVES v6.0 server (https://saves.mbi.ucla.edu/, accessed on 16 May 2024) [53]. The best tertiary structure model was selected based on Ramachandran plot analysis and ERRAT scores. Finally, the selected model was evaluated for potential errors using the ProsA server (https://prosa.services.came.sbg.ac.at/prosa.php, accessed on 22 May 2024) [54]. The final vaccine model was selected based on Ramachandran plot analysis, ERRAT scores, and ProsA z-scores.

### 2.9. Prediction of Conformational B-Cell Epitopes

B-cell epitopes are categorized as linear or discontinuous, with linear epitopes being more prevalent. Precise prediction of B-cell epitope conformations is essential for enhancing the spatial configuration of prospective vaccinations. The Ellipro tool (http://tools.iedb.org/ellipro/, accessed on 23 May 2024) available on the IEDB web server was employed for this purpose, as it can predict both linear and discontinuous epitopes [55]. The tool employs three primary algorithms to predict discontinuous epitopes. These algorithms categorize protein residues based on the prominence index (PI) and their spatial proximity, while allocating scores to epitopes according to the PI of their residues. Binding scores generated by ElliPro range from 0 to 1, with values closer to 1.0 indicating higher confidence in prediction. In line with standard practice, a threshold score of ≥0.5 was considered acceptable for downstream analysis. Using the tool’s default parameters, discontinuous B-cell epitopes with scores over the threshold were found and integrated into vaccine design.

### 2.10. Molecular Docking of Toll-like Receptors (TLRs) with Multi-Epitope Vaccines

Efficient immune responses rely on the proper binding of antigens or vaccines to target immune cells. To assess this, molecular docking analysis was conducted to evaluate the interaction between MEBPV and bovine immune receptors (TLRs). Protein structure data for bovine TLR2 (AlphaFold: AF-Q95LA9-F1), TLR3 (AlphaFold: AF-Q5TJ59-F1), and TLR4 (AlphaFold: AF-Q9GL65-F1) were obtained from the AlphaFold protein structure database (https://alphafold.com/, accessed on 13 May 2024) and the Protein Data Bank (PDB). The HADDOCK server (https://rascar.science.uu.nl/haddock2.4/, accessed on 13 May 2024) was employed to dock the MEBPV protein with TLR-2, TLR-3, and TLR-4 receptor proteins [56,57]. Interacting residues were identified and visualized using the PDBsum service (https://www.ebi.ac.uk/thorntonsrv/databases/pdbsum/Generate.html, accessed on 15 May 2024) [58].

### 2.11. Molecular Dynamics Simulation

The structural dynamics and flexibility of the MEBPV were assessed using the iMODS web server (http://imods.Chaconlab.org/, accessed on 20 May 2024). This server employs Normal Mode Analysis (NMA) to illustrate the coordinated motions of protein complexes within an intramolecular coordinate system [59]. Essential evaluation parameters include B-factors, deformability, covariance matrices, elastic network models, and eigenvalues.

### 2.12. Immune Simulation

An immunological simulation was conducted utilizing the C-ImmSim server (https://kraken.iac.rm.cnr.it/C-IMMSIM/, accessed on 21 May 2024) to assess the vaccine’s immunogenicity in the host [60]. The simulation was executed over 1050 iterations, with each iteration corresponding to 8 h. Vaccine injections were administered at intervals of 1, 63, and 126, corresponding to days 0, 21, and 42, respectively. In accordance with the server’s default settings, the antigen was delivered via the parenteral route, and each injection contained a standardized dose of 1000 antigenic molecules per volume. The simulations were conducted with a fixed random seed (seed = 12345) and the simulation was repeated three times (*n* = 3) to assess variability. Results are presented as mean ± SD.

### 2.13. Codon Optimization and in Silico Cloning

The vaccine design underwent reverse translation and codon optimization utilizing the online JCat tool (https://www.jcat.de/Start.jsp, accessed on 24 May 2024) for the E. coli K12 host strain [61]. The GC content% and codon adaptation index (CAI) were assessed to measure transcription and translation efficiency. Elevated CAI values and an ideal GC content range of 30% to 70% signify improved expression of exogenous genes [62]. Furthermore, *Bam*HI and *Xho*I restriction sites were added at the N- and C-termini of the cDNA, respectively, to facilitate cloning into the pET-28a (+) vector using SnapGene software.

## 3. Results

### 3.1. Protein Selection

The amino acid sequences of the HN protein (ALS46557.1) and F protein (ALS46556.1) of bovine parainfluenza virus were retrieved from the National Center for Biotechnology Information (NCBI) database (https://www.ncbi.nlm.nih.gov/)(Table S1). HN and F were selected due to their essential roles in viral entry, immune recognition, and previous experimental validation as protective antigens in paramyxoviruses [63,64]. VaxiJen analysis indicated antigenicity scores of 0.53 (HN) and 0.47 (F), both exceeding the viral protein default threshold of 0.4, thereby classifying them as antigenic. Both proteins were also predicted as non-allergenic, supporting their inclusion in downstream epitope mapping (Table 1).

### 3.2. B-Cell and T-Cell Epitope Screening and Evaluation

B-cell and T-cell epitopes of the bovine parainfluenza virus type 3 (BPIV3) HN and F proteins were identified using a combination of immunoinformatics tools, including IEDB, ABCpred, NetMHCIIpan, VaxiJen, and IFNepitope. The final selection of epitopes was based on a comprehensive set of criteria, including high antigenicity, strong binding affinity (IC50 ≤ 50 nM for T-cell epitopes), favorable percentile rank (≤1), and the predicted ability to induce IFN-γ responses, while ensuring non-allergenicity and non-toxicity. To achieve broad immune coverage in cattle, epitopes showing conservation across at least five of seven representative BPIV3 strains and the capacity of binding to multiple bovine MHC alleles were prioritized. This integrative strategy aimed to balance immunodominance and epitope diversity, ensuring the inclusion of epitopes that collectively provide wide immunological coverage rather than relying solely on individual antigenicity scores (Figures S1 and S2).

As a result, four B-cell epitopes and nine T-cell epitopes (five MHC-I and four MHC-II) were selected and incorporated into the multi-epitope vaccine construct (Table 2, Table 3 and Table 4).

### 3.3. Vaccine Design and Construction

**Figure 2:**
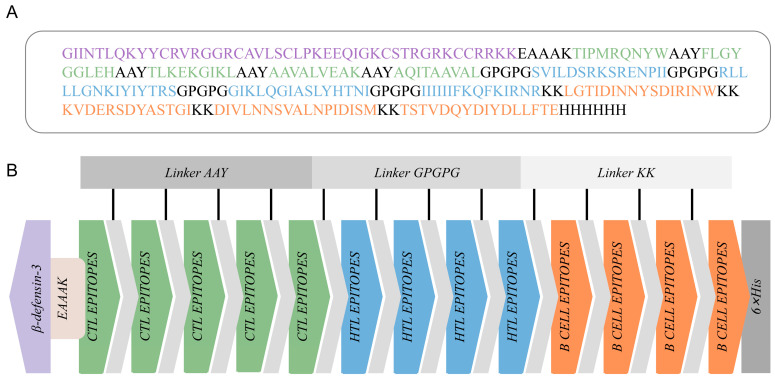
Construction of the multi-epitope-based peptide vaccine (MEBPV). (**A**) Amino acid sequence of the vaccine construct, highlighting CTL epitopes (green), HTL epitopes (blue), B-cell epitopes (orange), linker peptides (gray), β-defensin-3 sequence (light purple), and the 6×His tag (gray). (**B**) Schematic diagram of the vaccine construct.

The final MEBPV construct, illustrated in Figure 2A, comprises 5 CTL epitopes, 4 HTL epitopes, and 4 LBL epitopes, totaling 263 amino acid residues. This number was chosen to maximize epitope coverage of both HN and F proteins while minimizing redundancy, as overlapping or excessive epitopes may not enhance but instead skew immune responses. As shown in Figure 2, we connected these epitopes using AAY, GPGPG, and KK linkers, respectively. The AAY linker functions as a linker for MHC class I molecules, the GPGPG linker serves as a linker for MHC class II molecules, and the KK linker acts as a linker for B lymphocytes [65]. Additionally, the EAAAK linker was used to attach β-defensin-3 to the initial CTL epitope. Although β-defensin-3 has not been extensively studied in cattle, it has demonstrated efficacy as an adjuvant in various studies due to its immunomodulatory and antimicrobial characteristics [66].

### 3.4. Prediction of Vaccine Antigenicity, Allergenicity, and Toxicity

The antigenicity of MEBPV was predicted using the VaxiJen v2.0 server, yielding a score of 0.82, which indicates strong antigenicity. Both AllergenFP and AllerTOP classified the vaccine as non-allergenic, and the ToxinPred server confirmed its non-toxic nature. The data support the MEBPV design as a secure and efficacious candidate vaccine for BPIV3.

### 3.5. Physicochemical Properties of MEBPV

The physicochemical characteristics of proteins are essential to their functionality and immunogenicity, especially in vaccine development. The ProtParam tool was employed to examine the physicochemical properties of MEBPV. The vaccine has a molecular weight of 29.08 kDa, facilitating efficient absorption and processing by antigen-presenting cells (APCs), hence enhancing antigen presentation via MHC molecules. Theoretical isoelectric point (pI) is 9.81, signifying a positive charge at physiological pH, hence promoting contact with negatively charged immune cell membranes, including dendritic cells. The protein’s instability index of 21.95 categorizes it as stable (values beyond 40 denote instability), which is crucial for preserving structural integrity during storage and administration.

MEBPV comprises 41 positively charged and 19 negatively charged residues. The anticipated half-lives are 30 h in human reticulocytes, exceeding 10 h in E. coli, and above 20 h in yeast, indicating substantial stability across several systems. The aliphatic index of 95.40 indicates elevated thermal stability, facilitating adaptability across diverse temperature ranges. A GRAVY value of −0.27 signifies a hydrophilic characteristic, facilitating efficient interaction with solvent molecules and antigen transport. A solubility prediction score of 0.60 corroborates its appropriateness for formulation and distribution throughout biological systems.

In summary, the physicochemical properties of MEBPV highlight its stability and suitability for delivery, making it a promising candidate for a multi-epitope vaccine (Table 5).

### 3.6. Analysis of the Secondary and Tertiary Structures of the Vaccine

**Figure 3:**
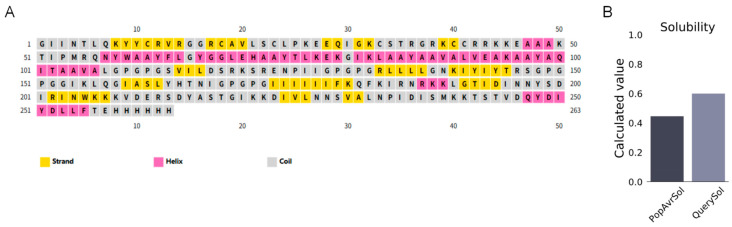
Secondary Structure Prediction and Solubility Assessment of the MEBPV. (**A**) Secondary structure prediction of multi-epitope vaccines using the PSIPRED tool. (**B**) Solubility prediction of multi-epitope vaccines performed with the ProtSol server.

The secondary structure of MEBPV was analyzed using the PSIPRED tool, indicating that the vaccine consists of 63 amino acids (23.96%) constituting α-helices, 59 amino acids (22.43%) comprising extended β-sheets, and 141 amino acids (53.61%) creating disordered coils (Figure 3A). The solubility score obtained from the ProtSol server analysis was 0.60 (Figure 3B), indicating favorable solubility properties.

**Figure 4:**
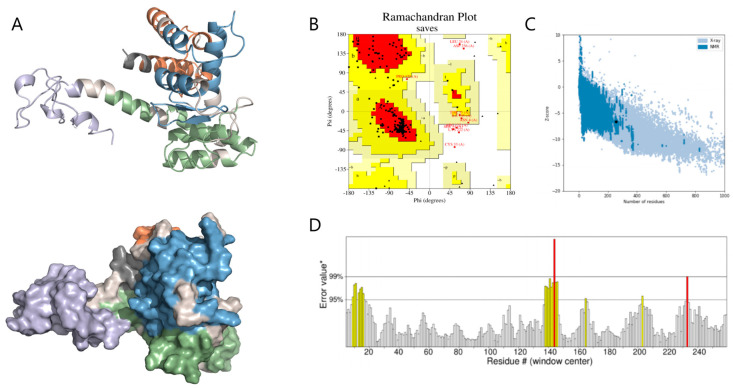
Predicted Tertiary Structure and Structural Quality Assessment of MEBPV. (**A**) The tertiary structure of the multi-epitope vaccine is depicted as a cartoon representation and surface view. In this visualization, the adjuvant is shown in light purple, the linker in light gray, the HTL epitopes in blue, the CTL epitopes in green, and the B-cell epitopes in orange. (**B**) Ramachandran diagram. (**C**) ProSA model quality assessment. (**D**) ERRAT diagram.* indicates the reference error value on the ERRAT score plot used for evaluation.

Tertiary structure prediction was performed using the Robetta server (RoseTTAFold pipeline; PDB templates updated to 14 June 2024), followed by refinement with GalaxyRefine v2.0 using default parameters. The final model was selected based on energy minimization and structural compactness. Structural analysis indicates that B-cell epitopes are predominantly located on surface-exposed protrusions, whereas most CTL and HTL epitopes are found on helices and loop regions. Each structural domain appears to effectively present its respective epitopes. Ramachandran analysis indicated that 85.7% of residues are situated in the most favored regions, 10.30% in additionally allowed regions, 1.30% in generously allowed regions, and 2.70% in prohibited regions, implying a generally dependable backbone geometry (Figure 4B). The MEBPV model exhibited a Z-score of −6.63 (Figure 4C) and an ERRAT score of 92.51 (Figure 4D), further supporting the predicted structural reliability and overall quality of the construct.

### 3.7. Prediction of Conformational B Cell Epitopes

**Figure 5:**
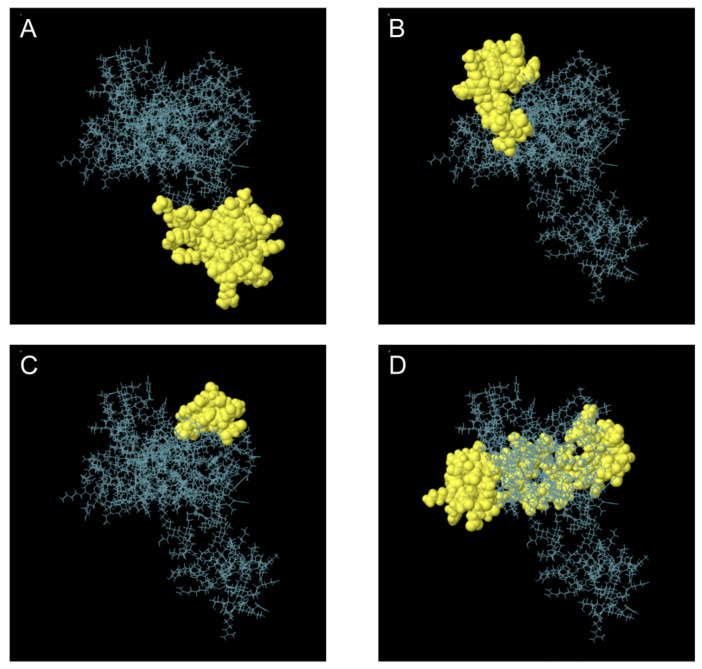
Three-Dimensional Representation of Discontinuous B Cell Epitopes in the Multi-Epitope Vaccine. (**A**–**D**) Discontinuous B cell epitopes are highlighted in yellow, while the remaining polypeptide regions are depicted as blue bars.

A total of 134 residues were identified as components of the expected discontinuous B cell epitopes. The lengths of these epitopes varied from 11 to 59 residues. The maximum binding score was 0.80 for a 41-residue epitope, while the second highest value of 0.68 was associated with a 26-residue epitope (Figure 5 and Table 6). In this context, binding scores closer to 1.00 are generally considered to represent stronger or more reliable epitope predictions, whereas lower values indicate weaker confidence.

### 3.8. Multi-Epitope Vaccine and Molecular Docking with Bovine TLRs

**Figure 6:**
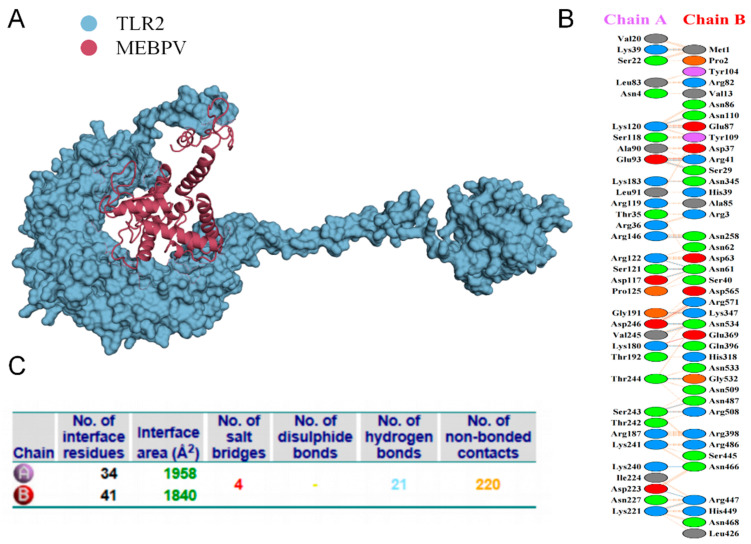
Docking of MEBPV with Bovine TLR-2 Molecules. (**A**) The docking complex between bovine TLR-2 and MEBPV. (**B**) Interacting amino acids at the interface of MEBPV (chain A) and TLR-2 (chain B). (**C**) Analysis of bond types formed within the docking complexes.

**Figure 7:**
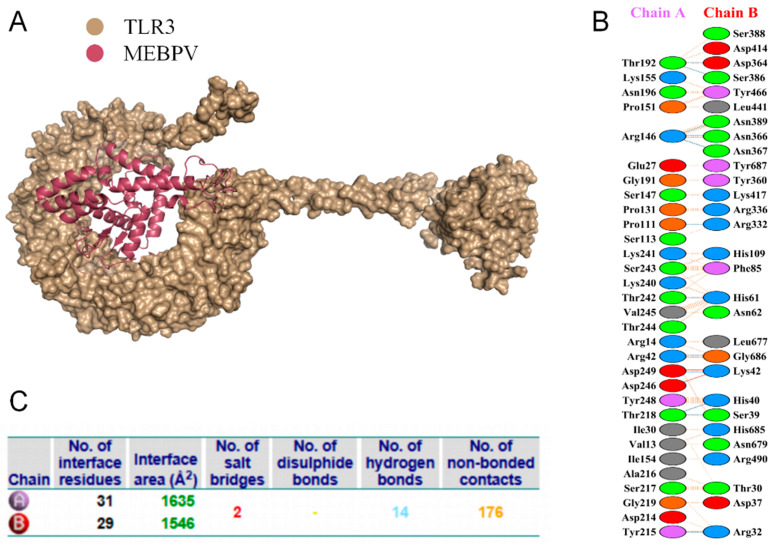
Docking of MEBPV with Bovine TLR-3 Molecules. (**A**) The docking complex between bovine TLR-3 and MEBPV. (**B**) Interacting amino acids at the interface of MEBPV (chain A) and TLR-3 (chain B). (**C**) Analysis of bond types formed within the docking complexes.

**Figure 8:**
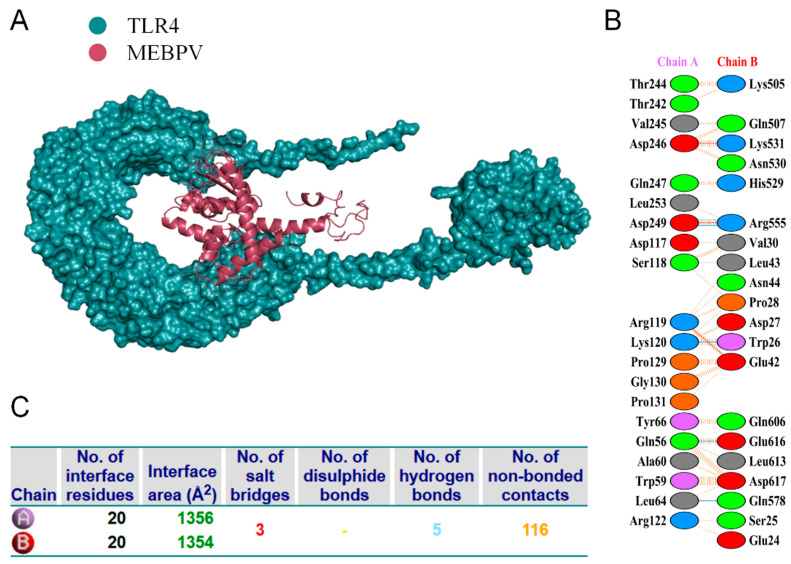
Docking of MEBPV with Bovine TLR-4 Molecules. (**A**) The docking complex between bovine TLR-4 and MEBPV. (**B**) Interacting amino acids at the interface of MEBPV (chain A) and TLR-4 (chain B). (**C**) Analysis of bond types formed within the docking complexes.

Proper binding between immune receptor molecules and antigenic targets is essential for the activation of immune responses. Therefore, in this study, molecular docking analyses were conducted between the MEBPV and Toll-like receptors TLR-2, TLR-3, and TLR-4 using the HADDOCK v2.2 server. Previous studies have demonstrated that TLR2 and TLR4 can induce antiviral immune responses by recognizing viral capsid proteins [45,67], while TLR3 effectively triggers immune activation upon virus recognition. TLR4 mediates immune responses by recognizing viral surface proteins [27]. Molecular docking analyses revealed high interactions between the MEBPV and these TLRs. The docking scores were 673.1 ± 16.5 for the TLR2-MEBPV complex, 613.5 ± 69.9 for the TLR3-MEBPV complex, and 639.6 ± 13.9 for the TLR4-MEBPV complex (Table 7). The binding interface analysis conducted using the PDBsum server revealed 4 salt bridges, 21 hydrogen bonds, and 220 non-bonding contacts in the TLR2-MEBPV complex; 2 salt bridges, 14 hydrogen bonds, and 176 non-bonding contacts in the TLR3-MEBPV complex; and 3 salt bridges, 5 hydrogen bonds, and 116 non-bonding contacts in the TLR4-MEBPV complex (Figure 6, Figure 7 and Figure 8).

### 3.9. Molecular Dynamics Simulations

**Figure 9:**
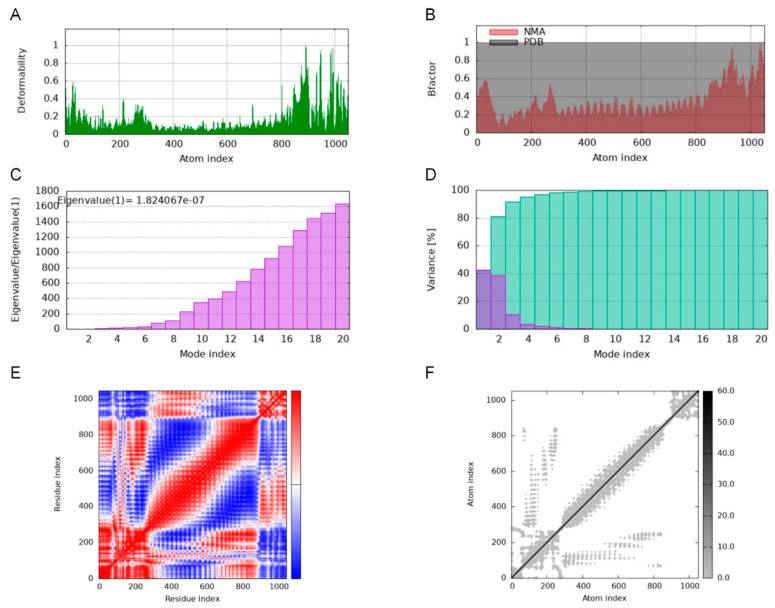
Molecular Dynamics Simulations of MEBPV-TLR2 Complexes. (**A**) Deformability analysis plot. (**B**) B-factor analysis plot. (**C**) Eigenvalue distribution plot. (**D**) Structural variance plot. (**E**) Covariance matrix plot. (**F**) Elastic network model plot.

**Figure 10:**
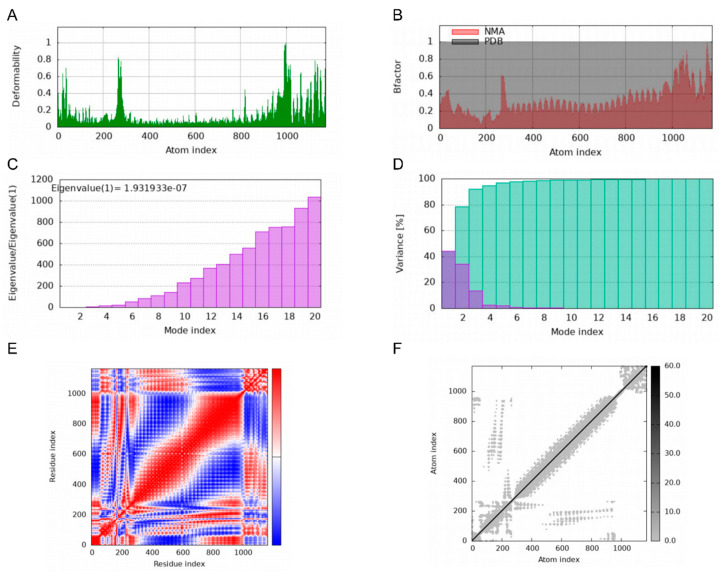
Molecular Dynamics Simulations of MEBPV-TLR3 Complexes. (**A**) Deformability analysis plot. (**B**) B-factor analysis plot. (**C**) Eigenvalue distribution plot. (**D**) Structural variance plot. (**E**) Covariance matrix plot. (**F**) Elastic network model plot.

**Figure 11:**
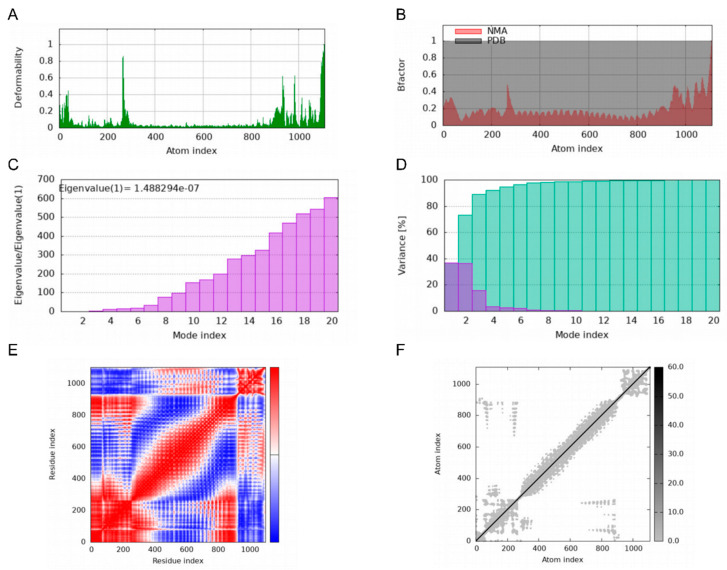
Molecular Dynamics Simulations of MEBPV-TLR4 Complexes. (**A**) Deformability analysis plot. (**B**) B-factor analysis plot. (**C**) Eigenvalue distribution plot. (**D**) Structural variance plot. (**E**) Covariance matrix plot. (**F**) Elastic network model plot.

Deformability plots suggested minimal deformations in the MEBPV-TLR2, MEBPV-TLR3, and MEBPV-TLR4 complexes (Figure 9A, Figure 10A and Figure 11A). B-factors, obtained from PDB data and normal mode analyses (NMAs), were employed to investigate the correlation between the molecular flexibility of the docked complexes and their corresponding PDB scores (Figure 9B, Figure 10B and Figure 11B). Eigenvalue plots illustrated the relative modal stiffness of the vaccine-TLR complexes (Figure 9C, Figure 10C and Figure 11C), with eigenvalues of 1.824067e-07, 1.931933 × 10^−7^, and 1.488294 × 10^−7^ for the MEBPV-TLR2, MEBPV-TLR3, and MEBPV-TLR4 complexes, respectively. Lower eigenvalues generally indicate that less energy is required for conformational deformation, reflecting potential molecular flexibility. Thus, while the values suggest that all three complexes retain stable binding interactions, the relatively lower eigenvalue of the MEBPV-TLR4 complex reflects comparatively higher conformational flexibility. Variance plots quantitatively depicted the contribution of normal modes to the overall dynamics of the protein complexes, with purple indicating individual variance and green representing cumulative variance (Figure 9D, Figure 10D and Figure 11D). Covariance plots highlighted the correlated motions of atoms within the complexes, with red regions showing correlated motions, white regions showing uncorrelated motions, and blue regions showing anti-correlated motions (Figure 9E, Figure 10E and Figure 11E). From a biological perspective, correlated motions may be associated with cooperative conformational changes relevant for receptor function, whereas anti-correlated regions may indicate structural tensions within the complex. The flexible network model was employed to analyze the rigidity of the TLR-vaccine complexes, identifying stiffer regions in dark grey and flexible regions as light dots (Figure 9F, Figure 10F and Figure 11F). The studies indicate that the vaccine-receptor complexes exhibit reasonably compact conformations with little oscillations; nonetheless, the somewhat unstable TLR3 interaction necessitates careful interpretation.

### 3.10. Immunological Simulation

**Figure 12:**
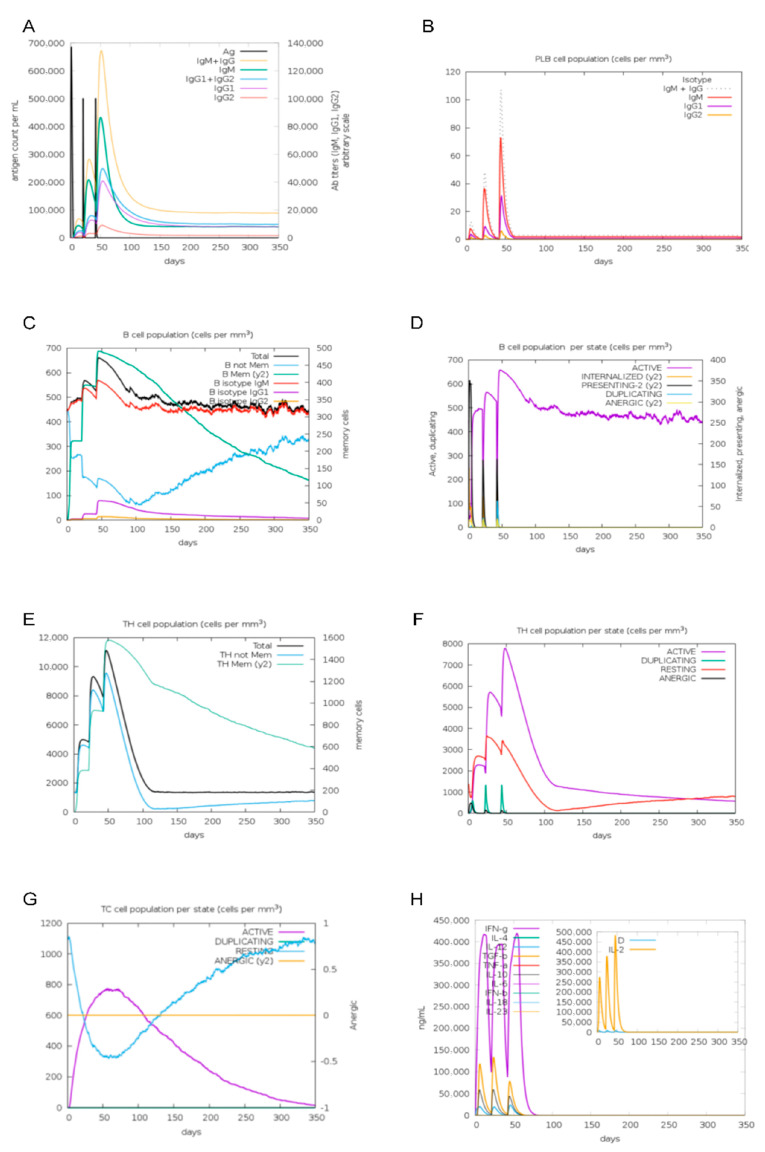
Immunological Simulation Analysis. (**A**) Immunoglobulin response to MEBPV vaccine. (**B**) Changes in plasma cell (PLB) populations. (**C**) B-cell populations. (**D**) Number of B cells per state. (**E**) TH cell populations. (**F**) Number of TH cells per state. (**G**) Number of TC cells per state. (**H**) Concentrations of interleukins and cytokines.

Immunological simulations using C-lmmSim were performed to predict the immune response in a cow model. The simulations indicated that antibody titers for IgG1 + IgG2, IgM, and IgM + IgG were significantly elevated following the second and third MEBPV vaccinations (Figure 12A). Additionally, the number of plasma cells (PLBs) expressing IgM and IgM + IgG isotypes increased markedly after the second and third immunizations (Figure 12B). Each vaccination led to a notable increase in the overall B-cell count and the quantity of activated B cells (Figure 12C,D). Furthermore, the MEBPV vaccine substantially enhanced the total number of helper T (TH) cells and activated T cells (Figure 12E,F), while activated cytotoxic T cells displayed a progressive increase following each vaccination (Figure 12G). Notably, the MEBPV vaccine significantly upregulated the expression levels of interferon-gamma (INF-γ) and interleukin-2 (IL-2) (Figure 12H). The data indicate that the MEBPV vaccine may elicit a good immune response against BPIV3.

### 3.11. Codon Optimization and in Silico Cloning

The MEBPV construct underwent codon optimization for expression in Escherichia coli, yielding a Codon Adaptation Index (CAI) of 0.81 and a GC content of 52.60%, signifying its suitability for the host’s codon usage; nonetheless, codon optimization by itself may not guarantee proper folding or functional expression of eukaryotic epitopes in E. coli.

**Figure 13:**
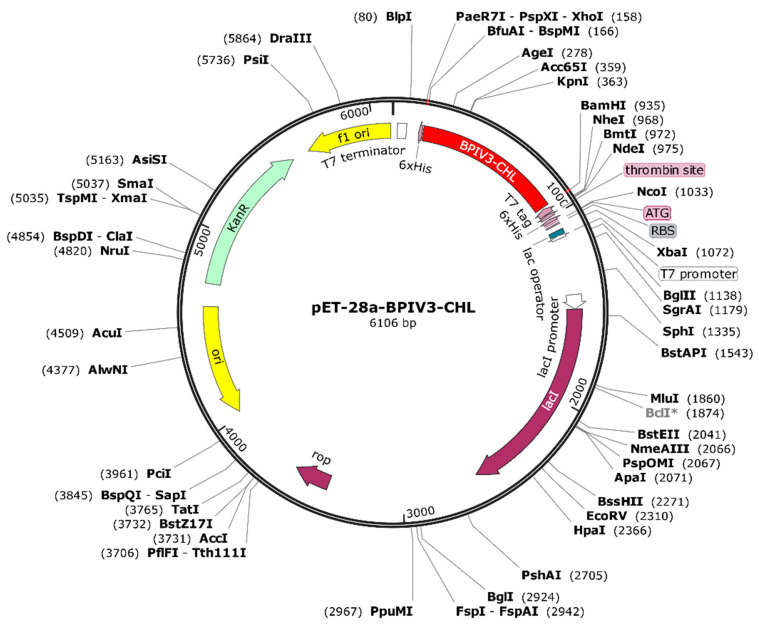
Plasmid Construction of the Codon-Optimized Vaccine in the *E. coli* K12 Expression System. The inserted DNA sequence is highlighted in red.BclI indicates a site flagged by SnapGene as potentially affected by DNA methylation or sequence context; plasmid DNA prepared from dam-/dcm- *E. coli* is expected to be cleavable at this site *.

A C-terminal 6×His tag was included for purifying convenience. Before any in vivo immunization, the His-tag will be enzymatically broken using a particular protease to reduce the risk of neo-epitope development and immune interference. The optimized sequence was inserted into the pET-28a (+) vector using BamHI and XhoI restriction sites, resulting in a recombinant construct of 6106 bp(Figure 13). Although theoretically appropriate for bacterial expression, experimental validation is necessary to evaluate the solubility, proper folding, and functional activity of the produced protein.

Additionally, for proteins expressed in E. coli, endotoxin removal procedures will be implemented to reduce LPS contamination and mitigate potential immune confounding in future studies.

## 4. Discussion

Vaccination is an essential strategy for the prevention and management of bovine parainfluenza. While conventional vaccines demonstrate significant immunogenicity, they have considerable drawbacks, such as elevated production costs—largely due to the necessity for specialized manufacturing facilities—and potential hazards linked to incomplete inactivation or viral leakage [17]. Recent progress in immunoinformatics and computational biology has yielded novel solutions for vaccine development. Notably, reverse vaccinology, a bioinformatics-based approach, offers a more efficient and cost-effective alternative to traditional methods by optimizing target protein screening and vaccine design [68].

In this study, the HN and F proteins of BPIV3 were selected as target antigens based on both computational and biological considerations. Although their antigenicity scores predicted by VaxiJen (0.53 for HN and 0.47 for F) were above the default viral protein threshold of 0.40, they represent moderate rather than high values when compared with other viral antigens commonly prioritized for vaccine development (e.g., >0.6 in RSV antigens). Despite this, HN and F were chosen because they play indispensable roles in viral attachment and membrane fusion, making them critical for viral entry and immune recognition. Furthermore, prior experimental studies in paramyxoviruses have demonstrated their capacity to induce protective immune responses, providing additional justification for their selection as vaccine targets. Highly antigenic, non-toxic, and non-allergenic T-cell and B-cell epitopes were identified using immunoinformatics tools and assembled into multi-epitope-based peptide vaccines (MEBPVs) with AAY, GPGPG, and KK linkers. These linkers help preserve the proper spatial arrangement of individual epitopes, ensuring correct folding and efficient, independent presentation to the immune system [69]. Additionally, the vaccine’s immunogenicity was enhanced by attaching β-defensin-3, a naturally occurring antimicrobial peptide, to the N-terminus as an adjuvant. β-defensin-3 activates antigen-presenting cells (APCs), thereby significantly enhancing the vaccine-induced immune response [70].

Toll-like receptors (TLRs) are crucial pattern recognition receptors (PRRs) that play a central role in innate immunity by recognizing conserved pathogen-associated molecular patterns (PAMPs) from various microorganisms. TLR2 and TLR4 can initiate antiviral immune responses by detecting viral capsid proteins [45,67]. TLR2 specifically identifies glycoproteins B and H of human cytomegalovirus and the E protein of SARS-CoV-2, initiating the synthesis of pro-inflammatory cytokines [71,72]. TLR3 efficiently elicits immune responses upon the recognition of viruses. TLR4 identifies various viral glycoproteins, such as the F fusion protein of Respiratory Syncytial Virus (RSV), the GP glycoprotein of Ebola Virus (EBOV), and the NS1 protein of Dengue Virus (DENV) [68,73,74]. Accordingly, TLR2, TLR3, and TLR4 were chosen for molecular docking with the engineered vaccine constructs. The binding stability and immunogenic potential of the vaccine were further validated through molecular docking and immune simulation. The immune simulation results demonstrated that MEBPV markedly increased the levels of IgG, IgM, IFN-γ, and IL-2, signifying its ability to enhance both cell-mediated and humoral immune responses.

In addition, the optimized vaccine DNA sequence was cloned into the pET-28a (+) vector, a favored prokaryotic expression system for recombinant protein production due to its high yield, rapid expression, low cost, and ease of purification [75,76]. The purified proteins require formulation with adjuvants or encapsulation in suitable delivery systems to enhance their stability, bioavailability, and immunogenicity. Subsequent studies should focus on determining the optimal dosage and immunization protocols, as well as further validating the safety and protective efficacy of the vaccine candidates through both in vitro and in vivo experiments [77].

This study has several limitations. It does not address whether epitope masking or misfolding within the recombinant construct might affect immune presentation, nor does it evaluate bovine population coverage. These factors restrict the generalizability of the findings and preclude definitive conclusions regarding broad-spectrum vaccine efficacy at this stage. In summary, this work applied a reverse vaccinology approach to design and preliminarily assess a multi-epitope peptide vaccine targeting BPIV3. The strategy offers valuable insights for the development of efficient, safe, and cost-effective vaccines. Nonetheless, the results are computationally predicted and derived solely from in silico analyses. Challenges may still arise during production and practical application, and the predicted immunogenic potential may not fully reflect actual in vivo efficacy, particularly regarding protective and cross-protective responses. Rigorous in vitro and in vivo studies are essential to determine the safety, immunogenicity, and protective efficacy of the proposed construct before it can be advanced as a viable vaccine candidate.

## 5. Conclusions

In conclusion, this study presents the in silico design and preliminary immunoinformatics characterization of a multi-epitope peptide vaccine targeting the HN and F glycoproteins of bovine parainfluenza virus type 3 (BPIV3). The construct exhibited favorable antigenic, physicochemical, and structural properties, suggesting its potential as a computationally supported vaccine candidate. Although computational simulations provide valuable insights, experimental validation is indispensable before practical application. Therefore, future work will focus on in vitro assays—including BoLA binding, antigen-presenting cell (APC) activation, and Toll-like receptor (TLR) reporter analyses—to confirm antigen–immune system interactions and evaluate the true immunogenicity of the designed construct. The study will also examine the augmentation of peptide stability, transport efficiency, and immunostimulant properties through nanocarrier formulations, Fc-fusion systems, or sustained-release adjuvants to augment efficacy in potential in vivo applications.

Notwithstanding inherent constraints, the amalgamation of bioinformatics and immunoinformatics technologies offers a robust and economic framework for the rational design of vaccines. Their increasing reliability in epitope prediction, structural modeling, and receptor interaction analysis is advancing veterinary vaccine research and may aid in the future development of next-generation vaccinations against BPIV3.

## Figures and Tables

**Table 1:** Details of target proteins.

Protein	GenBank	Amino Acid	Antigenicity Score	Allergen
HN	ALS46557.1	572	0.53	Non-Allergen
F	ALS46556.1	540	0.47	Non-Allergen

**Table 2:** Screening results of B-lymphocyte dominant epitopes of BPIV3.

Protein	Epitopes	Position	Amino Acids Number	Prediction Score	Antigenicity Score	Toxic	Allergen
F	TSTVDQYDIYDLLFTE	245–260	16	0.85	0.84	Non-Toxin	Non-Allergen
	DIVLNNSVALNPIDISM	442–458	17		1.23	Non-Toxin	Non-Allergen
HN	LGTIDINNYSDIRINW	434–449	16	0.94	1.55	Non-Toxin	Non-Allergen
	KVDERSDYASTGI	273–285	13		1.35	Non-Toxin	Non-Allergen

**Table 3:** Screening results of MHC-I dominant epitopes of BPIV3.

Protein	Epitopes	Position	Binding Alleles	Prediction Score	Percentile Rank	Antigenicity Score	Toxic	Allergen
F	AQITAAVAL	126–134	BoLA-D18.4	0.88	0.02	0.67	Non-Toxin	Non-Allergen
			BoLA-HD6	0.79	0.12			
			BoLA-1:00901	0.56	0.22			
			BoLA-T5	0.50	0.08			
			BoLA-1:00902	0.50	0.08			
	AAVALVEAK	130–138	BoLA-T2a	0.84	0.05	1.40	Non-Toxin	Non-Allergen
	TLKEKGIKL	220–228	BoLA-HD6	0.93	0.04	0.80	Non-Toxin	Non-Allergen
HN	TIPMRQNYW	398–406	BoLA-2:00801	0.50	0.14	1.25	Non-Toxin	Non-Allergen
	FLGYGGLEH	334–342	BoLA-1:00901	0.72	0.11	1.27	Non-Toxin	Non-Allergen

**Table 4:** Screening results of MHC-II dominant epitopes of BPIV.

Protein	Epitopes	Position	Binding Alleles	Prediction Score	IC50	Percentile Rank	Antigenicity Score	IFN-γ	Toxic	Allergen
F	GIKLQGIASLYHTNI	224–238	BoLA-DRB3*0301	0.72	15.09	2	0.92	0.24	Non-Toxin	Non-Allergen
			BoLA-DRB3*0303	0.90	2.44	0.5				
			BoLA-DRB3*0701	0.60	45.92	3				
			BoLA-DRB3*0901	0.64	32.26	4				
	IIIIIIFKQFKIRNR	509–523	BoLA-DRB3*0201	0.72	15.46	1.5	1.14	1.2	Non-Toxin	Non-Allergen
			BoLA-DRB3*0701	0.61	40.78	2				
			BoLA-DRB3*1101	0.86	3.88	1				
			BoLA-DRB3*1301	0.72	14.35	5				
HN	RLLLLGNKIYIYTRS	410–424	BoLA-DRB3*0101	0.60	48.13	16	0.42	0.76	Non-Toxin	Non-Allergen
			BoLA-DRB3*0201	0.62	38.97	6				
			BoLA-DRB3*1101	0.71	15.6	16				
			BoLA-DRB3*1301	0.71	16.8	6				
			BoLA-DRB3*0303	0.77	9.55	9				
	SVILDSRKSRENPII	492–506	BoLA-DRB3*0101	0.61	42.47	16	1.12	0.07	Non-Toxin	Non-Allergen

**Table 5:** Physicochemical Properties, Antigenicity, Allergenicity, and Toxicity Evaluation of the Vaccine Constructs.

Property	MEBPV
Number of amino acids	263
Molecular weight	29078.87 Da
Theoretical pI	9.81
Formula	C_1307_H_2107_N_373_O_361_S_8_
Estimated half-life	30 h (mammalian reticulocytes, in vitro). >20 h (yeast, in vivo). >10 h (Escherichia coli, in vivo).
Grand average of hydropathicity (GRAVY)	−0.27
Instability index	21.95
Aliphatic index	95.40
Antigenicity	0.82
Allergenicity	Non-allergenic
Toxicity	Non-toxic
Solubility	0.60

**Table 6:** Discontinuous B-cell epitope residues and scores.

No.	Residues	Number of Residues	Score
1	A:G1, A:N4, A:T5, A:Q7, A:K8, A:Y9, A:Y10, A:C11, A:R12, A:V13, A:R14, A:G15, A:G16, A:R17, A:C18, A:A19, A:V20, A:L21, A:S22, A:C23, A:L24, A:P25, A:K26, A:E28, A:Q29, A:I30, A:G31, A:K32, A:C33, A:S34, A:T35, A:R36, A:G37, A:R38, A:K39, A:C40, A:C41, A:R42, A:R43, A:K44, A:K45	41	0.80
2	A:H71, A:A72, A:A73, A:Y74, A:T75, A:L76, A:K77, A:E78, A:K79, A:G80, A:I81, A:K82, A:L83, A:A84, A:A85, A:Y86, A:A87, A:V89, A:A90, A:V92, A:E93, A:A94, A:A96, A:A106, A:L107, A:G108	26	0.68
3	A:I237, A:K240, A:K241, A:T242, A:S243, A:T244, A:V245, A:D246, A:Q247, A:Y248, A:D249	11	0.66
4	A:I115, A:L116, A:D117, A:S118, A:R119, A:K120, A:S121, A:R122, A:E123, A:N124, A:P125, A:I126, A:I127, A:G128, A:P129, A:G130, A:P131, A:T145, A:R146, A:S147, A:G148, A:P149, A:G150, A:P151, A:F182, A:K183, A:I184, A:R185, A:N186, A:L190, A:G191, A:T192, A:I193, A:D194, A:I195, A:N196, A:N197, A:Y198, A:S199, A:D200, A:I203, A:N204, A:K207, A:K208, A:D210, A:E211, A:R212, A:S213, A:D214, A:Y215, A:A216, A:S217, A:T218, A:G219, A:I220, A:K221, A:K222, A:N228, A:S229	59	0.64

**Table 7:** Molecular Docking Analysis of TLRs-MEBPV Receptor Complexes.

Parameters	TLR2-MEBPV	TLR3-MEBPV	TLR4-MEBPV
HADDOCK score	673.1 ± 16.5	613.5 ± 69.9	639.6 ± 13.9
Cluster size	2	4	8
RMSD from the overall lowest-energy structure	2.5 ± 0.4	16.9 ± 0.0	13.5 ± 0.7
Van der Waals energy	−75.9 ± 11.7	−86.8 ± 8.5	−90.1 ± 8.9
Electrostatic energy	−400.2 ± 157.5	−409.4 ± 44.6	−297.1 ± 34.6
Desolvation energy	11.5 ± 9.0	−0.1 ± 1.1	−18.5 ± 0.6
Restraints violation energy	8175.4 ± 177.1	7823.7 ± 634.5	8076.4 ± 189.8
Buried Surface Area	3598.8 ± 353.0	3387.1 ± 128.7	2915.6 ± 195.8
Z-Score	−1.5	−1.0	0.0

## Data Availability

The original contributions presented in this study are included in the article. Further inquiries can be directed to the corresponding author.

## Supplementary Materials

The following supporting information can be downloaded at: https://www.mdpi.com/article/10.3390/vetsci12111074/s1, Figure S1: Predicted immune epitopes mapped on the F protein of seven representative BPIV3 strains.; Figure S2: Predicted immune epitopes mapped on the HN protein of seven representative BPIV3 strains. Table S1: Comparison of genomic and HN/F protein sequence identity (%) among seven representative strains.
