# Supplementary material for: Multi-Epitope-Based Peptide Vaccine Against Bovine Parainfluenza Virus Type 3: Design and Immunoinformatics Approach
Source: Vet Sci. 2025 Nov 9;12(11):1074. doi: 10.3390/vetsci12111074 (PMC12656934; doi:10.3390/vetsci12111074)
Supplement: Supplementary file 1 [file vetsci-12-01074-s001.zip › vetsci-3920306-supplementary.pdf]

Table S1. Comparison of genomic and HN/F protein sequence identity (%) among seven representative strains.

Item	SD0835 (HQ530153.1)	XJ20055-3 genotype C (OM632676.1)	NX4 (OP718794.1)	Shipping Fever (AF178655.1)	BN-CE (AB770485.1)	XJ21032-1 genotype B (ON081628.1)	TVMDL17 (KJ647286.1)
**NX49 Genome (KT071671.1)**	99.26	99.60	99.63	82.38	82.10	81.04	81.39
**HN protein (ALS46557.1)**	99.30	99.48	99.48	85.84	85.49	84.44	84.44
**F protein (ALS46556.1)**	99.44	83.52	99.81	88.15	86.85	99.81	84.44

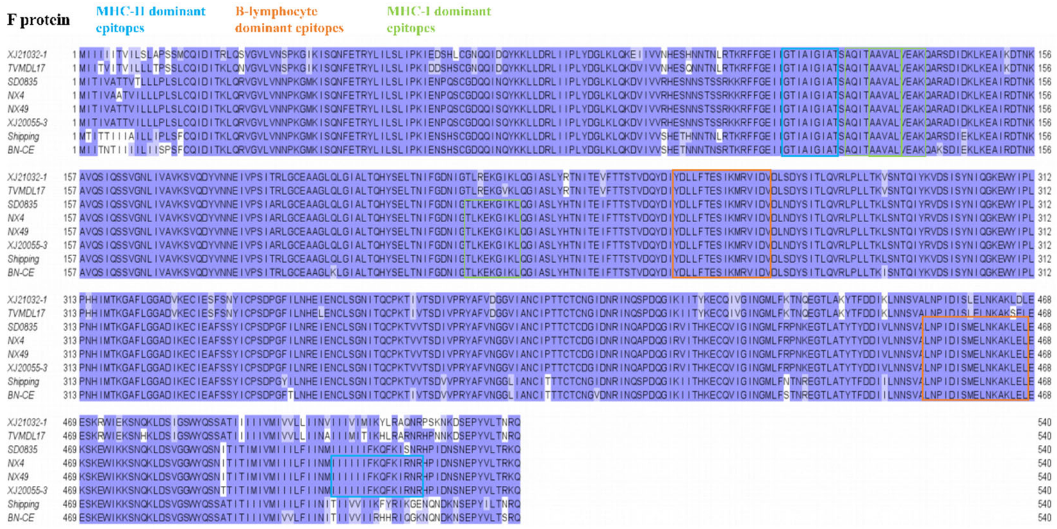

Figure S1. Predicted immune epitopes mapped on the F protein of seven representative BPIV3 strains. Epitopes are color-coded as follows: MHC-II dominant (blue), B-cell dominant (orange), and MHC-I dominant (green).

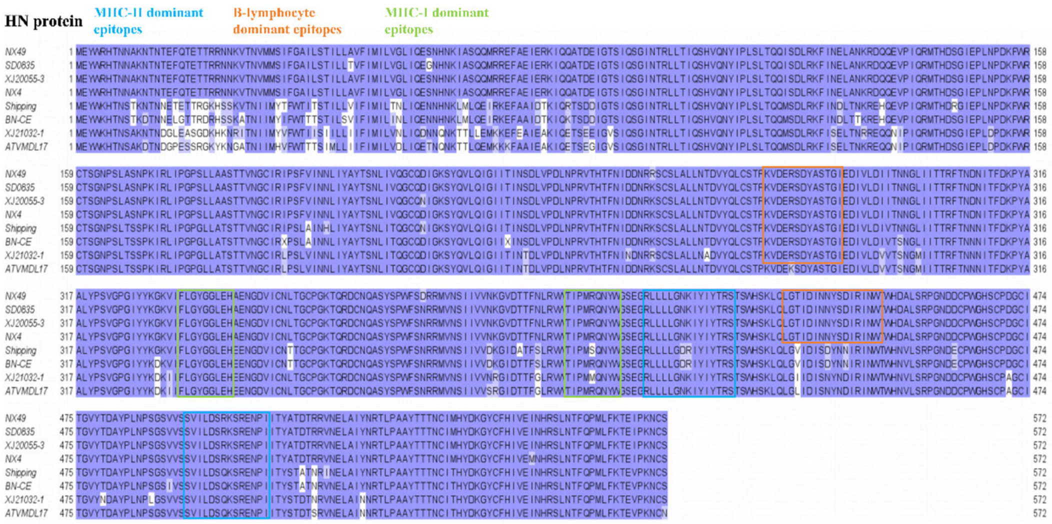

Figure S2. Predicted immune epitopes mapped on the HN protein of seven representative BPIV3 strains. Epitopes are color-coded as follows: MHC-II dominant (blue), B-cell dominant (orange), and MHC-I dominant (green).
